# The effect of family economy on mathematical problem-solving in fourth graders: the mediation of parental accompaniment and family book collection

**DOI:** 10.3389/fpsyg.2025.1723750

**Published:** 2026-01-29

**Authors:** Yan Yang, Na Zhang, Quan Zhang

**Affiliations:** 1 School of Mathematical Sciences, Guangxi Minzu University, Nanning, China; 2 Center for Applied Mathematics of Guangxi, Guangxi Minzu University, Nanning, China; 3 School of Education, Nanjing Normal University, Nanjing, China

**Keywords:** family book collection, family economy, fourth-grade students, mathematical problem-solving skills, parental accompaniment

## Abstract

Many Chinese parents experience anxiety stemming from their financial circumstances, limited time available for parental accompaniment, and insufficient cultural enrichment within the home. Fearing that these factors may adversely affect their children's academic achievements, they recognize that it is particularly crucial to investigate the influence of the family environment on a child's education. Mathematical problem-solving skills serve as a crucial indicator for assessing primary school students' academic achievement in mathematics and intellectual development. Using a questionnaire on family education from the Mathematical Problem-Solving Skills Assessment Survey as the measurement tool, the study targeted fourth-grade primary school students in a specific region of China and obtained 8,303 valid samples. Through correlation analysis and mediation effect testing, this study investigated the relationship between family economy parental accompaniment, family book collection, and pupils' mathematical problem-solving skills. Through regression analysis, the study highlights the impact of different forms of parental accompaniment on pupils' mathematical problem-solving skills. The findings revealed that: (1) Parental accompaniment, family economy, and the family book collection all predicted fourth-grade pupils' mathematical problem-solving skills. (2) Parental accompaniment and the family book collection partially mediated the influence of family economy on mathematical problem-solving skills. (3) Parental accompaniment significantly enhances pupils' mathematical problem-solving skills, with little difference in effectiveness between different forms of accompaniment. To this end, we believe that parental training programmes can help parents recognize the importance of quality time spent together and the value of a family book collection. Alternatively, providing resource support to families with limited financial means—such as book lending services or financial assistance—can optimize pupils' learning environments.

## 1 Introduction

Coleman (1968) point out that the most important factor influencing students' development is the family. The atmosphere of the family, the level of education of the parents, the economic situation of the family, the parents' commitment to their children's studies, the parents' expectations of their children, etc., affect the students' academic achievement to varying degrees (Cixian et al., 2023; Pinquart and Ebeling, 2020; Tárraga García et al., 2018). Families from different family economy backgrounds have different material and monetary investments in their children's education, as well as different investments of time and energy, thus family background affects students' academic achievement (Li and Qiu, 2018; Marks and Pokropek, 2019). For instance, the richness of home library resources, learning opportunities, parental availability, and the quality of time spent together all vary considerably. Within the Chinese cultural context, Chinese parents have higher academic expectations for their children (Cao et al., 2007) Chinese parents tend to be more involved than their Western counterparts, demonstrating greater willingness to participate in their children's learning and frequently communicating about school matters (Zou et al., 2013). Therefore, it is necessary to investigate the mechanism through which parental accompaniment and family book collection in China mediate the relationship between family economy and pupils' problem-solving skills in mathematics.

Students' mathematical problem-solving skills encompass the integrated application of multiple “key competencies” to resolve problems through the utilization of knowledge and skills. In terms of competency requirements, the mathematical problems students are expected to solve encompass comprehensive mathematical content domains and are grounded in mathematical problem-solving contexts relevant to their daily lives. Problem-solving skill encompasses model-based thinking, applied awareness, and innovative consciousness within core competencies. The fundamental characteristics of problem-solving assessment questions require that items be grounded in authentic contexts (real-life scenarios or genuine scientific problem-setting situations, excluding purely mathematical problem-setting contexts). This necessitates students undergoing a process of mathematical abstraction within the given context. Mathematical problem-solving skills encompass elements such as mathematical communication, reading, transformation, application, and the resolution of diverse problems (Lijun et al., 2023). It constitutes a vital component of mathematical literacy (ping, 2023).

In addition, the fourth grade of primary school (10–11 years old) is the “critical age” for the development of children's thinking, a key stage in the transition from concrete image thinking to abstract logical thinking. This stage, the ability of primary school students to formulate mathematical problems transitions from a lower level to a higher level (Chongde, 2008). Therefore, this study examines fourth-grade students in a district of a Chinese city, analyzing whether family economy and parental accompaniment significantly influence mathematical problem-solving skills. It further investigates whether parental accompaniment mediates the relationship between family economy and pupils' mathematical problem-solving skills.

## 2 Literature review and hypothesis

### 2.1 Problem-solving skills

There are many interpretations about problem solving in mathematics. Among these Polya opinion is the most referred by many math observers. Polya (2014) states that problem-solving is seeking a solution to a case that is not easy to solve. In the 2022 PISA assessment, mathematical problem-solving skills are defined as students' capacity for abstraction, representation, reasoning, computation, strategy or method design, knowledge, and reflection, which is primarily manifested in their ability to formulate, employ, and interpret mathematic (OECD, 2023). It is an important element of mathematical literacy (OECD, 2023). Students can be asked to apply mathematical modeling to tackle real-life problems. It is also very important to improve students' skills to solve a range of problems in their daily lives (Geiger et al., 2018). Exercising students' mathematical thinking, improving their procedural mastery and deepening their understanding of concepts can develop their mathematical problem-solving skills, which is conducive to their learning and lifelong development (Li et al., 2022; Santos-Trigo, 2024).

As an essential key skill for 21st-century learners (Partnership for 21st Century Learning (P21), 2015-0615), mathematical problem-solving competence is widely recognized as a core higher-order thinking skill among students. Its core essence refers to the cognitive analysis and practical problem-solving capabilities demonstrated by students when confronting mathematical problems in real-world contexts. Consequently, in recent years, international mainstream mathematics assessment programs [e.g., Programme for International Student Assessment (PISA), Trends in International Mathematics and Science Study (TIMSS)] have increasingly emphasized the weight of assessing this competence. The National Assessment of Educational Progress (NAEP) systematically evaluates students' level of mathematical problem-solving ability by assessing their mastery of skills in core domains such as number properties and operations, measurement, geometry, algebra, data analysis, statistics, and probability (Di et al., 2018). In China, some scholars have conducted specialized assessments of students' mathematical problem-solving proficiency by designing targeted test items for the compulsory education stage, based on the principles of consistency between students' problem-solving thinking processes, conclusions, and explanations, as well as practical rationality (Chunli, 2011). In summary, the core approach to measuring mathematical problem-solving ability currently lies in designing assessment content covering key areas such as algebra, geometry, statistics, and probability, thereby comprehensively evaluating students' comprehensive abilities in skill application and problem-solving.

### 2.2 The effect of family economy on pupils' mathematical problem-solving skills

Few researchers have examined the effect of family economy on pupils' mathematical problem-solving skills, but a large number of studies have shown that a family's finances are related to their children's mathematical learning and skills (Chiu and Chow, 2015; White, 1982; Yu et al., 2022). Moreover, considerable research has been conducted on the relationship between family economy and students' academic performance. Scholars contend that family economy exerts a certain effect on the academic performance of students across different age groups (Chunsheng et al., 2023; Gandarillas et al., 2024; Li et al., 2020). For instance, family socioeconomic status can significantly and positively predict secondary school students' academic performance (Li, 2023), and can also significantly and positively predict their performance in the secondary school admission examinations (Xiaosong et al., 2022). Niehues et al. (2020) found that family economy was significantly correlated with students' mathematics performance and exerted a significant effect (Wang et al., 2014). Amalina and Vidákovich (2023) believed that household income affects pupils' mathematical problem-solving skills. Concurrently, prior research suggested that schools and society should provide tailored support and intervention measures for students from different family economy backgrounds (Yongmei and Jing, 2021; Zhang et al., 2020).

However, some scholars contend that over the past few decades, the relationship between family socioeconomic status and academic achievement has gradually weakened, with the correlation being weaker for mathematics than for language subjects (Liu et al., 2020). Research has also indicated that low economic income may adversely affect academic performance, whilst high economic income does not necessarily directly promote improved results (Baoyan and Minggang, 2015), or may have negligible effects on specific groups (such as rural students; Shuangshuang, 2024). These inconsistencies hint that the influence of family economy may not operate directly, but rather requires explanation through more specific mechanisms. However, existing research has largely focused on examining the impact of family economy and sociolect-economic status on general academic performance or mathematics attainment. Less direct focus is placed on higher-order mathematical problem-solving skills—skills that emphasize strategic application, metacognition, and knowledge transfer. Moreover, few researchers have provided corresponding support and interventions for the impact of family economy on mathematical problem-solving skills. More importantly, there remains a lack of in-depth exploration into the specific mediating pathways through which family economy influences this ability. Therefore, moving beyond traditional “academic performance,” the focus shifts to the more complex “mathematical problem-solving skills”. This study, based on the characteristics of fourth-grade students, proposes the following hypothesis:

*H1:*Family economy can predict fourth graders' mathematical problem-solving skills.

### 2.3 The effect of parental accompaniment on pupils' mathematical problem-solving skills

There is no clear academic definition of parental accompaniment. Parental accompaniment broadly means that parents spend time with their children, planned or unplanned, in the family's daily life and provide material or moral support and assistance to their children in a positive parenting manner when necessary (Linyuan et al., 2017). For fourth-grade students (approximately 10 years old), who are at a critical stage in cognitive development and emotional regulation, appropriate education and guidance can significantly enhance their capacity for emotional self-control. Parental accompaniment is of paramount importance. Research has demonstrated that are parental accompaniment correlates with students' academic performance (Changfeng et al., 2021; Otani, 2020). However, there remains some controversy regarding the manner of companionship. On the one hand, different styles of parental accompaniment exert varying effects on children's academic performance. Parental emotional accompaniment positively effects children's academic performance, whereas recreational and study-focused accompaniment neither effectively promotes nor negatively impacts students' academic achievements (Zhengxiu et al., 2022). On the other hand, it is not necessarily the case that the more parental accompaniment children receive, the better their academic performance will be. For example, Li and Hamlin (2019) utilized data from the National Center for Education Statistics (NCES) to demonstrate that regular homework assistance can enhance student performance. Perna and Titus (2005) found that parental guidance and checking of students' homework had no significant effect on their academic achievement. On this basis, the impact of parental accompaniment methods on students warrants further investigation. More importantly, the majority of studies have focused on the impact of parental accompaniment on overall academic performance, without delving into specific subjects. Few researchers have investigated the impact of parental accompaniment on pupils' mathematical problem-solving skills. Based on this, the present study proposes the following hypothesis:

*H2a:* Parental accompaniment predicts pupils' mathematical problem-solving skills.

*H2b:* Different forms of parental accompaniment exert varying effects on pupils' mathematical problem-solving skills.

### 2.4 The mediating role of parental accompaniment between family economy and students' academic achievement

Family economy exerts an indirect effect on students by affecting a range of household activities. That is to say, it can indirectly affect students' academic performance through the family environment, parental involvement, parent-child communication, positive parenting practices, and parental expectations (Conger and Conger, 2002). For instance, Jinghuan et al. (2013) found that parental involvement mediated the relationship between family socioeconomic status and academic achievement among junior secondary school students. The quality of parent–child communication was positively associated with these children's academic (Yue, 2020). However, Leifeng and Jian (2017) found that the mediating effect of parental involvement on the relationship between family socioeconomic status and academic achievement was not significant. Family economy can exert an indirect influence on children's academic performance through the mediating effect of the home learning environment (soft learning environment, quantity of books held; Ye and Ze, 2023). Although some studies have examined the mediating role of parental involvement or family resources, research investigating the mediating effect of parental accompaniment and home library size between family economy and mathematical problem-solving skills remains relatively scarce. Based on this, the following hypothesis is proposed:

*H3a:* Parental accompaniment acts as a mediator in the process of family economy influencing mathematical problem-solving skills.

*H3b:* Book collection acts as a mediator in the process of family economy influencing mathematical problem-solving skills.

## 3 Tools

### 3.1 Samples

The data were derived from a survey of a district in one of China's new first-tier cities measuring mathematical literacy among fourth-grade students regarding their family backgrounds. The Mathematical Literacy Assessment Survey for primary school students was organized and arranged by the Education Department of the district. There were 45 elementary schools in the district, all of which participated in the test, with a total of 8,718 fourth-grade students taking the test and 8,718 papers being returned. After excluding the unanswered papers as invalid samples, 8303 valid samples were obtained, with a sample validity rate of 95.2%. Of these, 4,053 were boys and 4,250 were girls.

### 3.2 Pupils' mathematical problem-solving skills test paper

The mathematical problem-solving test for fourth-grade primary students was designed by key primary mathematics teachers and postgraduates, drawing on China's 2017 Compulsory Mathematics Curriculum Standards and the PISA 2021 Mathematics Assessment Framework. PISA 2021 emphasizes assessing students' ability to express, apply, and interpret mathematics in real-life contexts (personal, social, vocational, and scientific) as a key indicator of mathematical literacy, and its framework is widely recognized for effectively measuring mathematical problem-solving ability. The test papers were developed by Chinese experts in primary mathematics education and postgraduate students in primary education in accordance with the assessment framework, taking into account local characteristics and the developmental characteristics of primary school students. The test, tailored to China's primary mathematics education context and fourth-graders' cognitive development, consists of 10 units with 32 questions (single-choice, closed-ended, and open-ended). Question distribution covers number and algebra (38%), graphics and geometry (44%), and statistics and probability (19%).

### 3.3 Selection of family economy variables

Since family economy is mainly measured by income level, parents' education level, parents' occupation, family asset situation, social prestige and social relations. It is difficult for fourth-grade students to know accurately the real income of the family and the family's social prestige and social relations. Therefore, family economy in this study is measured by family asset status, which focuses on the actual status and ability of the family in terms of economy. Family economy was measured by the sum of the number of TVs, cars, computers and learning machines in the family for the students' family economy. Table 1 shows that 68.0% of the students' families have 1 TV, 68.1% have one car, 29% have one computer, 37% have two computers, and most of the students indicated that they do not have any learning machines at home. Additionally, we categorize students' family economy. According to Caner and Wolff (2004) asset weighting method, the following weights are assigned: —Motor vehicle (0.4)—Computer (0.3)—Learning machine (0.2)—Television (0.1). Threshold settings: Extreme poverty (total weight ≤ 0.3), Poverty (0.3 <total weight ≤ 0.45), Moderate (0.45 <total weight ≤ 0.65), Affluence (0.65 <total weight ≤ 0.85), Extreme affluence (total weight > 0.85). The majority of families have a modest family economy, with only a very small proportion being either extremely poor or exceptionally wealthy.

**Table 1:** Descriptive statistics of family economy indicators.

**Family resources**	**0 pieces**	**1 pieces**	**2 pieces**	**3 pieces**	**4 pieces and above**
Television	5.2%	68.0%	20.6%	4.5%	1.7%
Car	14.7%	68.1%	15.2%	1.5%	0.5%
Computer	4.9%	29.4%	37.2%	16.6%	11.9%
Learning machine	56.3%	36.6%	5.7%	0.7%	0.7%
Student family economy category	Extreme poverty	Poverty	Moderate	Affluence	Extremely affluence
	2.2%	20.7%	54.9%	19.6%	2.6%

### 3.4 Selection of parental accompaniment variables

The Parental Companionship Scale consists of four questions in total. Parental accompaniment in this study focused on the ways in which parents accompanied their children, including simple communication, accompaniment of learning in online classes, accompaniment of students in school activities, and accompaniment of students in interest activities. A more representative topic was chosen for each type of accompaniment. Parents' simple communication, accompanying students to school activities, and accompanying students to participate in hobby activities were as follows: “After school, mum and dad ask what you have learnt at school”, “Mum and dad take the initiative to participate in your school activities”, “Mum and dad do your favorite activities with you (e.g., walking, playing sports, going to the playground, etc.)”, and “Parents will accompany you to do your favorite activities (e.g., walking, exercising, going to the playground, etc.)”. The options for the first three questions were not at all (one mark), not match (two marks), unsure (three marks), compliant (four marks), and fully compliant (five marks). The title of the study companion was “What do your parents do when you are in class online?”. The options were: “Parents don't care if you go to class or not (one point); Parents do their own things and don't bother you in class (two points); Parents often come to see how you are doing in class (three points); Parents look up course materials for you (four points); Parents stay with you in class (five points)”. Higher scores indicate that students perceive higher levels of parental accompaniment. The reliability and validity analyses were conducted for the questions involved in parental accompaniment. The reliability coefficient for the theme of parental accompaniment was 0.625, which was acceptable (Taber, 2018). The Kaiser-Meyer-Olkin (Measure of Sampling Adequacy) (KMO) value for the topic of parental companionship was 0.678, with average validity, and the questionnaire could be used for subsequent analyses. Family Book Collection consisted of “How many books (excluding magazines) do you have in your household?”. The options were “0–25 books, 26–100 books, 101–200 books, 201–500 books, 500 +books”.

### 3.5 Data analysis

This study employed SPSS 27.0 for data analysis, encompassing the following sections. First, descriptive statistics were applied to the data using SPSS to characterize the current state of parental accompaniment and family book collection. Secondly, Pearson correlation analysis was employed to investigate the relationships between parental accompaniment, family economy, family book collection, and pupils' mathematical problem-solving skills. Thirdly, regression analysis was employed to investigate the effects of different accompaniment approaches on pupils' mathematical problem-solving skills. Finally, employing the Bootstrap method with 5,000 resampling iterations, we tested the significance of the mediating effects of parental accompaniment and family book collection between family economy and pupils' mathematical problem-solving skills.

## 4 Results

### 4.1 Descriptive statistics

The descriptive statistics of parental accompaniment were shown in Table 2. More than 90% of the parents asked what the students learned at school. About 90% of the parents actively participated in students' school activities and students' favorite activities. 45.6% of the parents did not disturb the students' online lessons, 33.5% of the parents regularly watched the students' online lessons, 3.3% of the parents helped their children to find information, and 16.6% accompanied their children's online lessons. Very few parents do not care about their students' Internet lessons.

**Table 2:** Descriptive statistics for each variable of parental accompaniment.

**Parental accompaniment methods**	**Not at all**	**Not match**	**Unsure**	**Compliant**	**Fully compliant**
Simple communication	1.7%	2.4%	3.0%	41.8%	51.1%
Accompanying to school activities	2.3%	3.6%	5.0%	31.3%	57.8%
Accompanying in hobby activities	2.0%	2.5%	2.7%	39.8%	53.0%
	1 point	2 points	3 points	4 points	5 points
Online learning companion	1.0%	45.6%	33.5%	3.3%	16.6%

The volume of a family's book collection refers to the quantity and diversity of books within the home, excluding items such as fashion magazines. It is a comprehensive indicator of cultural capital, reflecting to some extent the investment families make in cultural resources. The descriptive statistics of Family Book Collections were shown in Table 3. 40.8% of the households had 26–100 books, 24.1 % had 101–200 books and 18% had 201–500 books.

**Table 3:** Descriptive statistics of family book collections.

**Book resources**	**0–25 books**	**26–100 books**	**101–200 books**	**201–500 books**	**500+ books**
Family Book Collection	8.5%	40.8%	24.1%	18.0%	8.6%

### 4.2 Correlation analysis between variables

In this study, the correlations between the variables were analyzed through Pearson correlation as shown in Table 4. According to the results, it can be seen that there was significant positive correlation between parental accompaniment, family economy, family book collection and pupils' mathematical problem-solving skills. However, online learning accompaniment was negatively correlated with mathematical problem-solving skills. Online learning accompaniment was not correlated with family economy and family book collection.

**Table 4:** Correlation of dimensions.

**Variable**	**Mathematical problem-solving skills**	**Family economy**	**Parental accompaniment**	**Family book collection**	**Simple communication**	**Accompanying to school activities**	**Accompanying in hobby activities**	**Online learning companion**
Mathematical problem-solving skills	1							
Family economy	0.121^***^	1						
Parental accompaniment	0.160^***^	0.284^***^	1					
Family book collection	0.225^***^	0.390^***^	0.541^***^	1				
Simple communication	0.083^***^	0.133^***^	0.674^***^	0.164^***^	1			
Accompanying to school activities	0.107^***^	0.179^***^	0.674^***^	0.122^***^	0.411^***^	1		
Accompanying in hobby activities	0.106^***^	0.147^***^	0.742^***^	0.220^***^	0.559^***^	0.491^***^	1	
Online learning companion	−0.042^***^	−0.012	0.674^***^	0.005	0.111^***^	0.091^***^	0.154^***^	1

^***^p < 0.001; no asterisk indicates *p* > 0.05 (Field, 2024).

### 4.3 Mechanisms of family economy influence on pupils' mathematical problem-solving skills

The regression coefficients were tested for significance by repeated sampling 5,000 times using the Bootstrap method. We obtained robust standard errors (SE) and 95% deviation-corrected confidence intervals (CI) for the parameter estimates, and the regression results were shown in Table 5. After we added the mediating variables of parental accompaniment and family book collection. Family economy can positively predict parental accompaniment (β = 0.417, *P* < 0.001). Family economy was able to positively predicted family book collection (β = 0.146, *P* < 0.001). Family economy was able to positively predicted students' mathematical problem-solving skills (β = 0.227, *P* < 0.05). Parental accompaniment and family book collection were able to positively predicted mathematical problem-solving skills (β = 0.816, *P* < 0.05 and β = 0.836, *P* < 0.05). Preliminary indications are that the higher the family economy, the more parental accompaniment, and the larger the family book collection, the better the pupils' mathematical problem-solving skills. The higher the level of parental accompaniment, the higher the mathematical problem-solving skills of the students. The greater the number of books in the family, the greater the mathematical problem-solving skills of the students.

**Table 5:** Pathways of family economy on pupils' mathematical problem-solving skills.

**Variable**	**Parental accompaniment**			**Family book collection**			**Mathematical problem-solving skills**		
	β	**SE**	**CI**	β	**SE**	**CI**	β	**SE**	**CI**
Family economy	0.417	0.009	0.399–0.435	0.146	0.002 0.	142–0.150	0.227	0.086	0.059–0.400
Parental accompaniment	–	–_−_	–	–	–	–	0.816	0.0746	0.670–0.962
Family book collection	–	–	–	–	–	–	0.836	0.321	0.205–1.460
	*R*^2^ = 0.199 *P* < 0.001			*R*^2^ = 0.361 *P* < 0.001			*R*^2^ = 0.0287 *P* < 0.005		

The values of β in the table are the unstandardized coefficients.

Based on these analyses, we further quantified the relationship between parental accompaniment styles and fourth-grade students' mathematical problem-solving skills. Regression analyses of parental accompaniment styles and mathematical problem-solving skills were conducted using SPSS 27.0 and the results are shown in Table 6. The effect of simple communication on pupils' mathematical problem-solving skills was not significant (*P* = 0.078 > 0.05). Online learning support had a significant negative association with students' problem-solving abilities (*P* < 0.001, β = −0.061). Participation in school activities significantly enhanced students' mathematical problem-solving skills (*P* < 0.001, β = 0.070). Accompanying students in engaging with interest-based activities also produced a positive association with mathematical problem-solving skills (*P* < 0.001, β = 0.068). Participating in students' school activities and accompanying them to activities of interest were two types of accompaniment that had roughly the same effect on pupils' mathematical problem-solving skills. However, owing to the small R-square value, it can only be said that different forms of parental involvement exhibit certain positive or negative correlations with pupils' mathematical problem-solving skills. Nevertheless, this effect may not yet be sufficient to predict or influence such outcomes.

**Table 6:** Regression models for mathematical problem solving.

**Variable**	**Unstandardised coefficient**		**Standardized coefficient β**	***t***	**Significance**	**VIF**
	***B***	**Standard error**				
(Constant)	63.84	1.087		58.706	<0.001	
Accompanying to school activities	1.138	0.207	0.070	5.498	<0.001	1.366
Accompanying in hobby activities	1.212	0.252	0.068	4.813	<0.001	1.669
Simple communication	0.438	0.248	0.024	1.765	0.078	1.511
Online learning companion	−0.844	0.151	−0.061	−5.582	<0.001	1.025
*R* ^2^	0.019					
*F*	40.558					
Durbin-Watson	1.976					

The results of the correlation analysis between the variables can only indicate the correlation between family economy, parental accompaniment, and family book collection and students' mathematical problem-solving skills. However, there is a lack of exploration of the mediating role between the above four variables.

**Figure 1:**
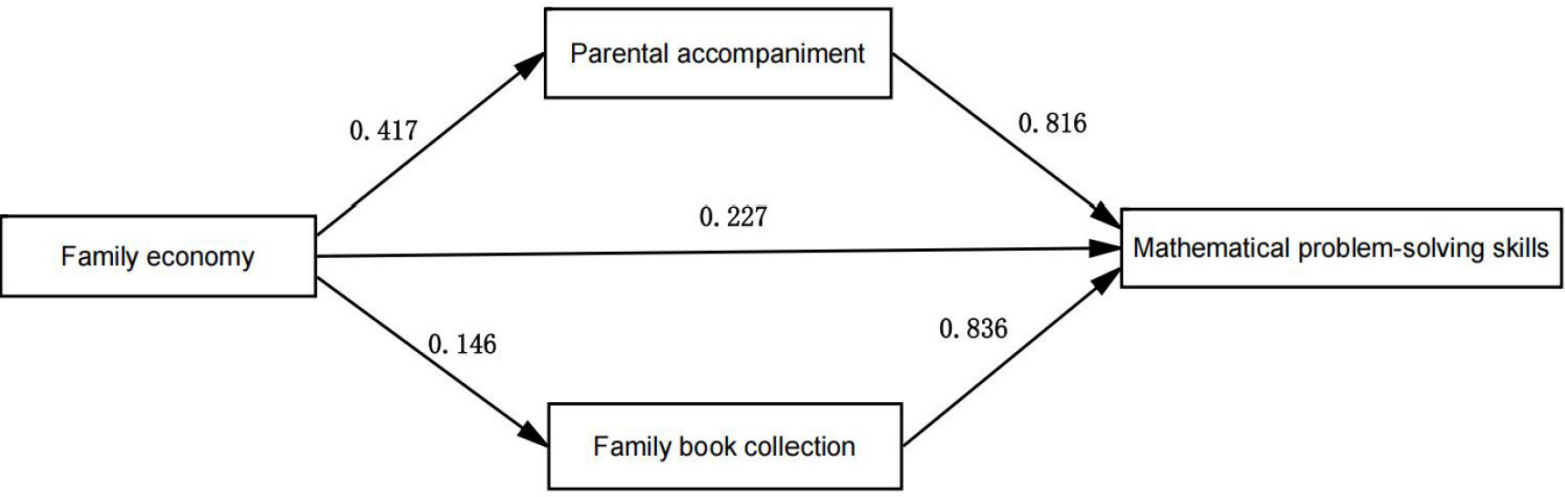
Diagram of the mediating effects model.

The significance of the mediating effect was tested using the Bootstrap method, with 5,000 replicates to derive 95% confidence intervals for the mediating effect, and the results were shown in Table 6. A model plot of the mediating effects of parental accompaniment and family book collection between family economy and students' mathematical problem-solving skills was shown in Figure 1. According to the results in Table 7, it can be seen that parental accompaniment partially mediated the effect of family economy on pupils' problem-solving skills in mathematics. The value of the indirect effect was 0.341 and the 95% confidence interval (0.271, 0.410) did not contain zero, thus indicating that the indirect effect holds. Family book collection partially mediated the effect of family economy on pupils' mathematical problem-solving skills. The value of the indirect effect was 0.122, which accounted for 49.36% of the total effect, and the 95% confidence interval (0.030, 0.216) did not contain zero, thus indicating that the indirect effect was valid. The direct effect value of the effect of family economy on pupils' mathematical problem-solving skills was 0.227, accounting for 17.69% of the total effect, and the 95% confidence interval (0.059, 0.40) did not contain zero. The direct effect was established.

**Table 7:** Analysis of mediating effects.

**Effect**	**Path description**	**Efficiency value**	**95 % CI**	**Relative effect percentage (%)**
Total effect		0.690	[0.567, 0.812]	100
Direct effect	Family economy → mathematical problem-solving skill	0.227	[0.060, 0.396]	32.97
Indirect effect	Family economy → parental accompaniment → mathematical problem-solving skills	0.341	[0.271, 0.410]	49.36
	Family economy → Family Book Collection → mathematical problem-solving skills	0.122	[0.030, 0.216]	17.69

## 5 Discussion

### 5.1 Factors affecting mathematical problem-solving skills

Firstly, regarding the direct effect of family economy on pupils' problem-solving skills, this study supports hypothesis H1. The findings indicate that family economy significantly predicts pupils' mathematical problem-solving skills. This conclusion aligns with previous research findings (Gandarillas et al., 2024; Li et al., 2020; Xiaosong et al., 2022). From the direct effect of parental accompaniment on pupils' problem-solving skills, this study supports hypothesis H2a. Research findings indicate that parental accompaniment holds predictive value for students' skills to solve mathematical problems, that is, the more involved the parents are with their children, the stronger the pupils' problem-solving skills. This conclusion is consistent with the findings of most scholars (Changfeng et al., 2021; Zhang et al., 2020).

Secondly, concerning the indirect effect of family economy on pupils' problem-solving skills, the study employs a mediation effect model. The results reveal that parental accompaniment acts as a mediator between family economy and pupils' skills to solve mathematical problems. Parental involvement plays a partial mediating role, supporting hypothesis H3a. The number of books in the household also has a mediating effect between family and pupils' problem-solving skills, supporting hypothesis H3b. The volume of books can be seen as a material investment in children's education, verifying Bradley's research conclusion that higher-income families are able to provide more resources for their children, thereby influencing students' academic performance (Bradley and Corwyn, 2002).

We have discovered that the family book collection has a significant positive effect on pupils' mathematical problem-solving skills. Moreover, the volume of books in the home partially mediates the relationship between family economy and pupils' mathematical problem-solving skills. The possible reason for this is that a family collection of books can directly and significantly positively affect students' reading ability. A family environment with a large number of books endows children with tools that are directly useful for school learning: vocabulary, information, comprehension ability, imagination, a broad historical and geographical perspective, and familiarity with good writing (Evans et al., 2010). Only with a relatively strong reading ability can pupils comprehend the context of questions on mathematical problem-solving skills, which constitutes a crucial step in successfully translating real-life problems into mathematical ones. Moreover, the family book collection reflects parents' commitment to education (Fan, 2021). Parents who prioritize education are likely to invest more in educational expenditure, while culturally enriched homes can better stimulate pupils' academic interest, enhancing children's cognitive abilities and academic performance. At the same time, children sense their parents' commitment to education, which can motivate their dedication to learning.

### 5.2 The effects of different accompaniment approaches on pupils' mathematical problem-solving skills differ only marginally

Although certain forms of parental accompaniment is related to students' mathematical problem-solving skills, they only explain a small portion of the variation in pupils' problem-solving skills. Therefore, parents need not agonize over which method to choose for parental accompaniment. Regarding the mechanism by which parental accompaniment styles influence pupils' mathematical problem-solving skills, it is evident that, apart from the negative association between having parents physically present during online lessons and pupils' mathematical problem-solving abilities. Several other distinct forms of parental accompaniment exert a positive relationship with pupils' mathematical problem-solving skills, though the impact of different accompaniment methods varies little. In other words, as long as parental accompaniment—whether accompanying children in sports or discussing school-related matters—has a broadly comparable impact on pupils' mathematical problem-solving skills. This may be because the presence of parents during children's online lessons is perceived by the children as a sign of mistrust, which is detrimental to fostering their independent learning habits and academic interest. Parental accompaniment, as witnessed by engaging in sports activities and school events with their children, and engaging in discussions about life and studies, helps create a harmonious family atmosphere, enabling children to feel valued by their parents and thereby enhancing their interest in learning and their commitment to their studies. The reason why these different forms of parental involvement yield similar effects may lie in the fact that parental accompaniment does not directly teach children mathematics, but rather enhances their engagement and interest in learning by conveying parental care and expectations.

Parental active involvement in their child's school activities has a positive correlation with the pupil's mathematical problem-solving skills. This may be because parental participation not only enables them to better understand the school's educational philosophy but also fosters collaborative relationships with teaching staff. Such engagement helps establish a more conducive learning environment for the child. When pupils perceive their parents' attention to their education, it enhances their motivation to study mathematics. Concurrently, parental accompaniment in enabling pupils to participate in leisure pursuits exerts a positive influence on their mathematical problem-solving skills. Engaging with children in activities such as jigsaw puzzles, sports, outings, and painting not only enriches their extracurricular experiences but also cultivates their concentration, logical reasoning skills, innovative thinking, and teamwork ethos. For instance, jigsaw puzzles can develop pupils' logical thinking skills, whilst activities such as drawing and craftwork can stimulate their creativity. Football, meanwhile, cultivates concentration and teamwork. These abilities indirectly influence pupils' mathematical problem-solving skills.

However, when students take online classes, parental accompaniment in their learning can have negative association with their mathematical problem-solving skills. This finding is similar to the view of Zhengxiu et al. (2022), who found that parental involvement in learning negatively impacts students' academic performance. This conclusion does not necessarily mean that parents' involvement in their children's learning is harmful. It could indicate that parents tend to become more involved because their children are struggling academically (Milne et al., 1986). In China, it is often the mother who helps students with their studies, and it's possible that the emotional dynamics between the mother and child during study sessions—such as the presence of negative emotions—may suppress the child's motivation to learn, ultimately affecting their academic achievements (Pomerantz et al., 2005). Additionally, students might perceive their parents' presence during online classes as an attempt to prevent them from engaging in non-academic activities, like playing games, which could provoke a rebellious response from the students.

In conclusion, this study finds that family economy, parental accompaniment, and the number of books in the home have a significant positive effect on students' mathematical problem-solving ability. Parental accompaniment and the number of books at home partially mediate the relationship between family economy and pupils' mathematical problem-solving skills. Simple communication does not significantly affect pupils' problem-solving skills. The four types of parental accompaniment discussed in this study contribute only a small part to the overall influence on pupils' mathematical problem-solving skills.

## 6 Revelation

In the process whereby family economy influences mathematical problem-solving skills, parental accompaniment and the family book collection exert a partial mediating effect. This implies that improving a family's economic circumstances not only directly promotes pupils' mathematical ability development, but also further optimizes the learning environment through indirect means such as increasing the quantity and quality of parental time spent with children and expanding the family's book collection. This indicates that the family environment plays a significant role in the development of students' learning abilities. Consequently, policymakers and providers of social resources should focus on how to indirectly enhance students' learning abilities by improving their families' family economy. Schools and educational institutions may develop corresponding interventions based on this. For instance, parental training programmes can help parents recognize the importance of spending time with their children and maintaining a family book collection. Alternatively, resource support such as book lending services or financial assistance may be provided to families with limited means, thereby optimizing pupils' learning environments.

## 7 Limitations

This study only explored the impact of family economy, parental accompaniment, and the number of books in the home on the mathematical problem-solving skills of fourth-grade students (aged 10–11). The results may not be applicable to students of other age groups. The sample for this study was drawn from a district within an emerging first-tier city in China, where the overall economic, cultural, and family book collection levels exceed the national average. Students in this district demonstrate overall academic abilities that exceed the national average. Consequently, the findings may not be applicable to pupils in China's second-tier and third-tier cities, nor to those in rural and ethnic minority regions. Furthermore, owing to cultural variations between nations, these research outcomes may not be transferable to other countries.

Moreover, the measurement tools present shortcomings: the low internal consistency reliability of the Parental Accompaniment Scale hinders the accurate reflection of the quality of parental involvement. Cognitive resources such as household books are measured using a single item, lacking multi-item validation and prone to measurement error. Furthermore, certain data pertaining to young children's cognitive experiences and learning attitudes rely on self-reporting by the children themselves. Such data may be influenced by the children's linguistic expression abilities, cognitive levels, and susceptibility to social expectations, potentially leading to self-reporting bias that could compromise the accuracy of the findings.

Finally, as this study is cross-sectional in nature, it is difficult to establish causal relationships. For instance, while families with higher economic standing may place greater emphasis on education, it cannot be ruled out that households enjoying exceptionally privileged financial circumstances might not prioritize their children's education to the same degree. Future research could expand sample sizes and incorporate longitudinal studies to explore more comprehensively the intricate relationship between family economy and pupils' academic achievement. This would provide a more scientifically grounded basis for the development of educational policies and the allocation of family educational resources.

## Data Availability

The original contributions presented in the study are included in the article/supplementary material, further inquiries can be directed to the corresponding author.
